# Looking Back

**Published:** 1934

**Authors:** H. Elwin Harris

**Affiliations:** Consulting Surgeon to the Bristol Royal Hospital for Sick Children and Women


### The Bristol Medico-Chirurgical Journal

*" Scire est nescire, nisi id me
Scire alius sciret."*

SPRING, 1934.

### LOOKING BACK.

The Presidential Address, delivered on 11th October, 1933, at the opening of the Sixty-first Session of the Bristol Medico-Chirurgical Society.

BY

H. Elwin Harris, M.A., M.B. Cantab., F.R.C.S. Eng.,

*Consulting Surgeon to the Bristol Royal Hospital for Sick Children and Women.*

First allow me to offer you my best thanks for electing me your President for this year. When the Secretary wrote to me that the Committee proposed to nominate me as the President-Elect, it was an agreeable and, I must admit, a most unexpected surprise. After the most careful introspection I have been unable to find anything in my past history which would justify the Committee's action. However, I will endeavour to the best of my ability to uphold the dignity and traditions of this Society, and trust I may prove a worthy representative of that great body of general practitioners for whom this honour is really intended.

On their behalf and my own I again thank you most sincerely.

To the majority of those who occupy this Chair the choosing of a subject and writing of an address presents little difficulty. The specialist in his speciality can always find some abstruse matter which he can elaborate, dilate upon, and when occasion requires use it to edify, perchance to bewilder, an audience. But to a man like myself, who has spent his whole life in practical work, writing up an address is no mean task, and it has been a source of many misgivings. As it is, I can only offer you to-night a talk which might be most aptly described as a thing of shreds and patches.

Sinking into my subconscious self in the hope of getting an inspiration for to-night, I found that there stood out far and above anything else the startling changes and discoveries which have taken place in every branch of medical knowledge since I commenced practice—changes and advances of such magnitude that it is hardly credible they have all occurred in one life-time.

I propose to enumerate many of these with some views and reminiscences of my own. I would then ask why all these remarkable discoveries were reserved until the end of the nineteenth century and the beginning of this. After that I would point out how our increased knowledge of the nature, treatment and prevention of infectious disease and pestilence has affected the population and mortality of the nation. And if I have time I should like to make a few remarks on the fact that in spite of our manifold and precise means of diagnosis and our more exact knowledge of disease, quackery in its divers forms, faith-healing and healing by fancy religions, is as rife as it was in the Middle Ages.

In all reforms and great changes there is always a pioneer, and in this case it was Joseph Lister—afterwards Lord Lister. In 1860 he was appointed to the Chair of Surgery at the Glasgow University and Surgeon to the Glasgow Infirmary. The younger generation of medical men here to-night, who were, as it were, born and brought up in an atmosphere of asepsis, will scarcely believe the condition of the patients in the surgical wards of the Glasgow Infirmary when Lister was appointed. We read that the Infirmary was a hotbed of sepsis. Healing by first intention was practically unknown; the mortality after the major operations was appalling. Suppuration, phagedæna and blood-poisoning carried off the most promising cases, and were the sequelæ after the most trifling operations. Hospitals which were founded for the welfare of the poor during the wave of philanthropy which swept over Europe in the eighteenth century were now dreaded by the very people for whom they were intended, and had become veritable death-traps. So desperate were the conditions that James Simpson wrote denouncing the mortality in the hospitals, and even suggested that the hospital system should be done away with altogether. Fortunately, Lister had already commenced his work, and was trying to get at the root of this fatal sepsis, which at that time claimed no less than 45 per cent. of his own amputations. He had already asked himself the question why a simple fracture never suppurated but a compound one almost invariably ended in death from sepsis and blood poisoning, and he had come to the conclusion that it must be due to something in the air which poisoned the wounds. Simultaneously a French chemist, Pasteur, was working on putrefaction, and he found that putrefactive fermentation was dependent upon living organisms which gained access to the putrescible material from the dust in the air, dust on surrounding objects, and from other putrefying media. Lister immediately applied this knowledge to his work in surgery, and saw that to avoid sepsis it was essential that germs should be prevented from getting into the wound during and after operation. And secondly, that if they were already in the wound some means to destroy them must be found before they obtained a foothold and multiplied. To destroy bacteria and germs Pasteur used boiling. This was obviously of no use to Lister: some chemical had to be found. His attention was called to the use of carbolic, which was then sold under the name of German Creosote, in the treatment of sewage. He obtained a sample of this, and this was the first antiseptic Lister used, and although in after years he was willing to give any other suggested to him a trial, as a matter of fact he preferred carbolic and remained true to it up to the day of his retirement.

In the year 1880 the British Medical Association held its annual meeting at Cambridge. I was then a student at the University and had scarcely commenced any medical study, but the chance of seeing the lions of the profession was too good to be missed, and I wangled leave to go to any of the sections I wished. On the first day I found myself, much abashed, sitting in the back seat in the surgical section, and the first speech I ever heard on any matter professional was one delivered that day by Joseph Lister on a general discussion of the treatment of wounds. Mr. William Savory of St. Bartholomew's Hospital, for whom incidentally I dressed three years later, was in the Chair.

Mr. Lister produced a number of stoppered bottles in which he had placed with antiseptic precautions a quantity of fresh bullock's blood some time previously, and which he had kept at a temperature of 99·5°. In some of these he had inoculated the blood with septic material, and to others he had added different quantities of rain water. As far as I could gather the great point he made was that the blood-clot, if undisturbed, had the power of protecting itself against the development of organisms. He attributed this power of resistance to the white corpuscles, but emphasized the great importance of covering up the wound and also of keeping the part at rest. The impression I received was that the majority of surgeons were opposed to his methods.

One surgeon, however, Mr. John Wood of King's College, who evidently had a radical cure for hernia, spoke very much in favour of antiseptic methods. He told us that he had performed 137 operations for radical cure of hernia in the previous twelve months after Mr. Lister's methods, many of the cases coming from America, and that he had not only had no deaths but no suppuration. But as I have said, the majority of surgeons were opposed to Mr. Lister's methods, and nothing could have expressed this feeling better than the final sentence of the speech of one of the leading surgeons:[1] "Until better results had been shown to follow antiseptic methods they were fully justified in continuing a practice which was the mature result of many years of experience." Quite extraordinary were the indifference, opposition, and even hostility to Mr. Lister's methods shown by some surgeons who occupied the highest positions in the profession.[2] And amongst these no one more than my chief, Mr. William Savory. When I dressed for him Lister had been at work on antiseptics for twenty years, and it was three years after Mr. John Wood had related his successes. Yet Mr. Savory was not perturbed that most of his operation cases suppurated, and he still pinned his faith on linseed and bread poultices. He was considered one of the most able men on the staff of St. Bartholomew's. He was certainly a brilliant lecturer: each one of his lectures was a polished oration. As he ignored antiseptics, so he had a contempt for drainage. When lecturing on this subject on one occasion he had before him a glass full of water and a piece of drainage tube of large calibre. After a most eloquent peroration, in which he carried on an argument between himself and an imaginary opponent, he said: "Gentlemen, if I were lecturing to a learned body of surgeons on this subject I should take this tube and place it in the glass of water. I would say: Gentlemen, observe nothing happens, the water does not leave the glass. But if I take the glass in my hand, still holding the tube in it, and turn the glass upside down, the water immediately flows on the floor and the glass is soon emptied. True, some of the water flowed through the tube, but the result would have been precisely the same had no tube been present." But in spite of opposition in England, antiseptic surgery gained ground on the Continent, where Listerism was far more appreciated than at home.

Neuber of Kiel was the first to practise boiling every instrument and dressing, the clothing of the patient and the overalls of the surgeon, assistants and attendants. This practice proved so satisfactory that it was soon adopted in most countries. In 1886 Schimmelbusch introduced the sterilization of dressings by super-heated system. In 1891 Halstead of Baltimore suggested the wearing of boiled rubber gloves. And in 1900 Hunter of Charing Cross started the wearing of masks, and aseptic surgery could then be said to be the finished article. This was exactly forty years after Lister had commenced his work in Glasgow.

Turning now to medicine. During the later half of the period we have been discussing, more particularly that decade 1880 to 1890, the acquisition of new knowledge with reference to and the discovery of the ætiology and the specific remedies of disease was amazing. And this period is and ever will remain, in regard to the saving of life and the benefit to humanity at large, the most wonderful in the history of medicine.

Pasteur's discovery that definite diseases could be produced by bacteria gave a stimulus to research in the ætiology of infective conditions. In 1876 Koch obtained the bacillus anthracis ; and the introduction by him in 1881 of solid culture media was most important, and led to a remarkable progress in the study of bacteriology. From 1880 to 1890 the bacteria of suppuration, tubercle, glanders, diphtheria, typhoid, cholera and tetanus were all identified and their relation to the individual diseases established. Those of plague and dysentery followed in the next decade, and also that of influenza according to some observers. Although these organisms were discovered, it took the profession some time to realize that such minute particles could be of any importance. I was present at a conversazione in London at which a tubercle bacillus was shown under the microscope shortly after its discovery. This exhibit was of such interest that it was necessary to queue up in order to see it. Immediately in front of me was a member of the staff of one of the large London hospitals, who after looking down the microscope turned to a friend and said: "One can hardly believe that anything so small can do so much harm!" That bacillus at that time was killing about forty thousand people a year.

Having found their organisms, the next question was to study how immunity to diseases produced by bacteria could be effected, and it was found that it could be accomplished by attenuated infection. In 1884 Metchnikoff proved his theory of phagocytosis. One of the most important investigations followed, viz. that of the toxic effects produced by the bacteria. This led to the inquiry into immunity against toxins, and the result was the discovery of the antitoxic action of the serum obtained from animals immunized against certain toxins such as those of tetanus and diphtheria. This, again, led to the introduction of one of the most valuable therapeutic agents of the age, viz. diphtheria antitoxin. It is now nearly forty years ago since the introduction of this most important and life-saving remedy, and it would be difficult for anyone now to realize the extraordinary boon that it was at that time. The mortality of diphtheria was something like 85 per cent., and it was not uncommon for two and sometimes three children in a family to die in two or three days, to say nothing of the agony the children suffered from slow suffocation. This remedy has practically abolished the operation of tracheotomy. Cases of diphtheria in those days were treated in the wards, and this operation fell to the lot of the House Surgeon. Occasionally an adherent plug membrane had to be removed by suction through a tube, which sometimes cost the operator his life. The next year saw Marmorek bring out the anti-streptococcic serum, which, though the earliest, has remained one of the most frequently used of that class of preparation. Widal's test and the demonstration by Sir Almroth Wright that vaccines injected induced a condition of active immunity after infection had occurred soon followed.

It is only necessary to mention the conquest of the tropics: the work of Manson, Ross and others on malaria, yellow fever and other diseases, which has so revolutionized the countries known only a few years ago as the White Man's Grave that they are now included in the list of places to be visited in the "round the world" pleasure cruises.

Another discovery of great interest and importance was that of the spirochæta pallida and the Wassermann reaction. The improvement in the modern treatment of syphilis has to a great extent robbed this disease of its horrors. Whether there are a large number of cases of syphilis still extant and whether the clinics absorb them I cannot say, but speaking from my own experience in practice during the last few years, syphilis appears to be very much on the decline. But it is to congenital syphilis and especially in regard to those cases in which it remains latent far beyond the ordinary accepted period of the first seven years of life to which I would refer.

Some years ago I read a paper before our district branch of the British Medical Association on a case of congenital syphilis in a girl of twenty-three. Both she and her mother affirmed that until three years before she had been in good health. After that she developed a stuffiness in the nose, some pain and soreness in her throat, and dysphagia and a swollen knee-joint. When I saw her she had most typical syphilitic disease of the nose with perforation of the septum and masses of purulent crusts. One-third of her epiglottis was eaten away, and she had a typical syphilitic knee-joint with melon seed bodies. I satisfied myself that this girl had not acquired syphilis, but the magic way in which her nose cleared up with liquor hyd. perchlor. and rapidly increasing doses of pot. iod. left no doubt about the diagnosis. When writing my paper and looking up the bibliography of the subject, I could only find three other cases on record of congenital syphilis in which the symptoms did not show themselves until the twentieth year. I am persuaded in my own mind that there must have been many more, and that when symptoms do not appear until late adolescence or early adult life some of these cases are entirely overlooked, especially those limited to the central nervous system. To illustrate what I mean I cannot do better than tell you the story of a pathetic domestic tragedy. I knew this case personally, but was in no way responsible for her professionally.

A young lady passed through her childhood and teens and reached the age of twenty-two. She had shown herself at school to be well above the average in intelligence, she was good-tempered and possessed an amiable disposition. Later, when assisting her mother in entertaining, she was a delightful hostess. In addition to all this, although it has nothing to do with it, she was one of the best horsewomen in her county. At the age of twenty-two she showed signs of an irritability and temper which hitherto had been foreign to her nature and which in course of a year or two became pronounced. Later she presented a new phase in her character, and would drive into the nearest town, and if she saw and took fancy to anything in a shop, such as a fashionable dress, totally regardless of the cost, she would purchase it and put it down to her father. On one occasion she bought a beautiful tea service of some expensive china. Her father was not of the best-tempered, and after the bills came in you can quite understand that the domestic atmosphere was somewhat blue. It never occurred to anyone around her that she might be suffering from some incipient disease, and there *was* some excuse for this, because in the intervals the girl could be quite charming. This is confirmed by the fact that a great friend of hers, who had known her for some years and who apparently saw no change in her disposition or manner, proposed to her and in due course married her. He little knew what he was letting himself in for, but he soon found out. After the marriage he was remarkably good to her, but she became absolutely impossible. To make the story short: after two years of married life she contracted a chill and was attended by a medical man. He does not appear to have ever suspected there was anything else the matter with her than the chill, and after attending her for a fortnight he advised her to go away for a change. The husband by this time was absolutely fed up, and he decided to leave her. He wrote a note in which he handed over to her the house and furniture, told her she was mad or bad, he did not know which, but that he would never live with her again. This was given her on her return. She made some attempts to win him back, but these were futile. She then attempted to plant herself on a married brother who had not the accommodation to spare, but when he refused to take her in she sent him the most insulting post cards. As he was rector of the parish, this was a matter which he could not ignore, and he gave in. However, she became rapidly worse, and at length was seen by a mental specialist, who immediately certified her, and she was removed to an asylum. She died three years after admission. Her brother fortunately insisted on a post-mortem. He was informed that she died from syphilitic disease of the brain. The membranes were so thickened and so contracted that the brain itself was to all intents and purposes annihilated. This case, I think, is sufficient to illustrate what I wish to impress upon you, that congenital syphilis may be and is sometimes entirely overlooked. It is also of great interest as showing the course run by congenital syphilis when untreated. In this particular case, had it been suspected, the diagnosis would have been quite easy. On inquiring into her family history it would have been found that her father had had syphilis, had been overtreated with mercury until every tooth in his jaws fell out and he lost every hair from his head, and he died one year after this girl was married of a ruptured aneurism of the aorta.

I had a very similar case in my own practice a few years ago, but I only mention it to warn you not to rely upon a Wassermann. Do not imagine that I wish to infer that every cantankerous triangular old maid is the subject of congenital syphilis. But amongst those people who are impossible to live with and who are on the border-line, I am sure that many would derive far more benefit from large doses of pot. iod. than from psycho-analysis.

In November, 1895, another discovery in science was made which has been of inestimable value in medicine and surgery. In November of that year Röntgen was experimenting with a Crookes's tube in the Department of Physics in the University of Würzburg. On shading it with his hand he was astonished to find that a shadow appeared showing distinctly the bones of his hand. This was the beginning of X-rays. I was present at the first lecture given on this subject by Sylvanus Thompson in 1896. At this lecture the X-rays were passed through a closed compass box, and the shadow of the set of compasses was seen on the screen; a man with a fish-hook in his leg, which had been there for twenty years, was also screened. To be able to see the shadow of the set of compasses through the closed box was considered something marvellous. But the actual skiagrams in those days were very crude. Owing to the weakness of the rays and the poor apparatus the exposure was necessarily very long, and in a medical journal of 1897 there is a statement that the exposures of an hour or more were trying to the patient and that the body heat tended to melt the gelatin off the photographic plate. Its value now as a help in diagnosis and in other ways is so well known that it is only necessary to mention it.

Amongst the list of invaluable new remedies may be mentioned radium, massage, remedial exercises, ionization and diathermy, ultra-violet and infra-red rays, and one other remedy which can be obtained by the just and the unjust without money and without price, but which has, in my opinion, done more to raise the standard of the nation's health than anything else, and that is "open air."

I cannot allow obstetrics to pass without saying a few words about them. Like many others, in my early days I had to use midwifery, unremunerative as it was, as a means to a practice. Young members of the profession to-day arrive at that goal through the much easier and more pleasant means of the Panel, and unattractive midwifery is left to midwives. During the past few years obstetrics have been very much in the limelight, and one is continually reading in the daily papers something about the dreadful mortality. All the remedies suggested to reduce this mortality appear to me to be directed to the elaboration of the surroundings of the case, and no emphasis is laid on the fact that the responsibility for this mortality rests with the attendance and after-attention of the case. The point I wish to stress to-night is that, in my opinion, surroundings have nothing whatever to do with maternal mortality. It was only the other day that I came across a new book by Munro Kerr on *Maternal Mortality and Morbidity.* I only had time just to dip into it, but the first thing which attracted my attention was a table of the mortality from 1855 to 1930.[3] From 1855 to 1864 the mortality per thousand of live births was 4·7, of which 1·6 were due to puerperal sepsis. From 1925 to 1930 the rate was 4·2, and that with a much lower birth rate, and of these 1·7 were due to puerperal sepsis. These figures are astonishing and require some explanation. The maternal mortality of the present day is only 0·5 less, whilst that from puerperal sepsis is actually 0·1 higher than it was in 1855. That is before the aid of antiseptics. This confirms what I have said: that surroundings have nothing whatever to do with maternal mortality, and also to prove this I will, in spite of the risk of being thought egoistical, relate my early experiences of midwifery.

When a student at St. Bartholomew's we were sent out into the slums to do the twenty confinements required for examination purposes, and the only qualification necessary was that we had read, marked, learned and inwardly digested a little book written by a Clifton doctor known as *Swayne's Aphorisms*. Out of my twenty I only had one difficult case, a very small woman with a contracted pelvis who had gone on to full time. Ante-natal clinics were unknown then. I allowed her to have some pain for a short time to see if anything would happen, but finding they were useless I fetched the midwifery assistant. The pelvis was so small and the child so large that he decided that it would have to be removed piecemeal. Now in these days this woman would have been removed to a hospital, she would have had a Cæsarean section, performed under the most up-to-date aseptic methods and in an operation theatre with all the latest appliances. Well, this little woman was operated upon in a small, dirty room in a typical London slum by the midwifery assistant in his shirt sleeves, and I gave the anæsthetic in my shirt sleeves and rendered him all the assistance I could. The operation lasted over three hours. Were I to relate this case to any of the thousands of people who are continuously reminded in the daily papers of the still lamentable maternal mortality they would at once exclaim: "Poor woman, of course she died!" Not at all. In a month she was about doing her ordinary work, and she caused me no anxiety whatever.

During the first few years I was in Clifton I did a few hundred cases of midwifery in the Hotwells and Clifton Wood. In very few, if any, of these had I a nurse who was entitled to call herself a trained nurse. I never allowed these people to have anything to do with the patients during confinement. I did everything myself, including the bathing. I did not leave the house until I was satisfied that the patient was, as far as one could judge, aseptic. Every one of these cases was visited for four consecutive days after the confinement, and off and on for a further fortnight at least. If there was any after-treatment to be done in the way of douching I always did it myself. The result was—I say it in no boastful spirit—I never lost a case, and only on about four occasions do I remember having any fever which caused me anxiety. In favour of my views I may quote from a little book published in 1773, written by a Dr. Charles White, a Fellow of the Royal Society, on the management of pregnant and lying-in women and the means of curing and more especially preventing the particular diseases to which they are liable.[5] He states there that he had never lost a case by puerperal sepsis. He attributed puerperal sepsis to the effects of mismanagement by the accoucheur or nurses, or else arising from the patient's own imprudence. Nothing could emphasize the importance of the personal element more than the experience of Semmelweiss of Vienna, who has been called the forerunner of Lister.[5] In the year 1846 Semmelweiss lost 459 cases out of a total of 4,010 healthy young women, that is to say, 11·4 per cent.[2] He found no satisfactory explanation of this mortality in such causes as overcrowding, fear, mysterious atmospheric influences, or even contaminated wards. Just about this time he lost a friend through a poisoned finger, and this suggested to him that in his cases the poison might be carried to the patient. So he insisted that every student, every attendant, every nurse should before they approached his patients in future thoroughly wash their hands in a solution of bleaching powder, no change being made in their surroundings. This alone reduced his mortality to 45 out of 3,536 cases, that is 1·2 per cent.

It has been said that a confinement being a physiological and not a pathological process, the element of carelessness is apt to creep in; if so, this is to be deplored. In discussing this matter with Dr. Askins, our late Medical Officer of Health, he told me that maternal mortality was greater than that of the major abdominal operations. Quite so, but if everyone attending a confinement went about it in precisely the same manner as a surgeon does a major abdominal operation the result would probably be different.

Having enumerated these remarkable discoveries in medical knowledge, one naturally asks why all this should have been reserved for the latter part of the nineteenth century. There are two reasons. One is the absence of the microscope, and the other is the late period at which the dissection of the human body was commenced.

With regard to the micro-organisms, it is obvious that their discovery was not possible until the invention of the modern microscope. Leeuwenhoek in the seventeenth century invented the one-lens microscope, by which only the largest micro-organisms and animalculæ could be seen. It was only when the compound microscope (which I believe I am right in saying was first invented by a spectacle maker, Jansen, a Dutchman, in 1590) and when to a very much improved model of this Abbe added his sub-stage in the early part of the latter half of the nineteenth century, and when water immersion lenses were invented by Hartnack, 1855, glycerine immersion by Grunlach, 1867, and the oil immersion lenses by Amici in 1869, that it became possible to identify the more minute of the bacteria.

The failure of the ancient physician to attain to our knowledge of the common diseases could not be attributed to a lack of genius. Has any genius ever excelled that of Hippocrates, Aristotle, Galen, and many others ? But whatever their genius, they could not arrive at any exact medical science without a fundamental knowledge of anatomy and physiology, and this was denied them because the dissection of the human body was forbidden. The Greeks disallowed it. The Egyptians certainly did some in a desultory manner, mostly following the disembowelling and embalming of the dead. During the Mediæval Ages the Arabs held it to be unholy and unclean to touch a dead body, and the Church forbade it ; consequently, after the time of Galen very little advance was made in medical science until the end of the Mediæval Ages. Galen dissected the ape, dog, ox and pig, and it was believed that the structures of the human body were identical with those of these animals until the time of Vesalius, 1543, who first seriously dissected the human body. Galen's views were very much respected by the Church because his belief was that all structures in the body were formed by the Creator for a known and intelligible purpose, and this view appealed both to Christians and Mohammedans alike. When Vesalius commenced to expose some of the errors of Galen the Church was very indignant, but Vesalius persevered and convinced them that the hip bones of man were smaller and narrower than Galen supposed. But the Church still maintained that Galen could not have been mistaken, but that the bones had changed since his time owing to men having taken to the fashion of wearing tight trousers.

But, apart from all serum and vaccine therapy, have we not gone back to the rational treatment of Hippocrates ? There were two hundred and fifty odd drugs known in his time, but he used very few of them, and relied on the healing power of nature, hygienic living, fresh air, rest, diet and regulated exercises and baths. Is this not to all intents and purposes the treatment of to-day ?

Our next question is : In what way has all this new knowledge of infectious disease, its treatment and prevention, affected the population and mortality of the nation? In order to appreciate this you must allow me to give you some details of the pestilences of ancient times. In those days disease and pestilences were regarded as an inevitable affliction of mankind, the visitation of the Almighty for the sins of the people. And this idea was encouraged by the Church and accepted by the people. The main diseases were: leprosy, typhoid fever, typhus fever, and the plague. But for terrible mortality the last was the most responsible.[6] The plague raged from time to time from A.D. 79 to 1665. In the year A.D. 164 it commenced in Rome and raged for sixteen years, and at its height killed as many as ten thousand people in one day. Again, in Constantinople the plague from A.D. 524 to 594 took a toll of one million lives. The epidemic which started in Africa and Asia in 1345 and two years later appeared in Constantinople, Italy, Greece and England, and was known as the Black Death, lasted with short intervals until 1655—a matter of three hundred years—and killed one-fourth of the population of the entire world. No one at this time ever dreamt of the real cause of the plague. It was not until those marvellous few years at the end of the nineteenth century that the blame was definitely put upon rats, and it was found that fleas leaving the dying rats infested man and gave him the disease. Had we not this knowledge, we should be as defenceless against the disease as the people in London in 1665.

It is obvious that in those days, when pestilence was continually decimating the population, to compensate for the prodigious mortality you must of necessity to keep up the average population have a prodigious birth rate. The necessity for this has long passed. Both our annual infant and other mortality is very low. All the recent discoveries in medicine must therefore tend to produce a surplus population. But this is not all. All these recent discoveries are used to foster the survival of the unfit.[7] In an interesting article written by Dr. A. F. Tredgold on eugenics in the *Cambridge University and Medical Society's Magazine* he calls attention to the fact that "in ancient times the severe struggle for existence was the natural condition of affairs, and that those who were better able to secure their food and escape being killed by other creatures survived and propagated their type." In other words, this was a survival of the fittest and led to a progressive evolution. "To aid this man in those days established a custom for children and adults who were defective or deformed in body or mind to be put to death or to be expelled from the community—which means the same thing. As civilization advanced we find firstly an accumulation of new knowledge, and secondly the growth of the humanitarian sentiment. The latter has led to the abolition of the putting to death of delicate infants and the establishment of ante-natal clinics, lying-in hospitals, midwifery services, infant welfare centres, school clinics, national health insurance, hospitals, sanatoria, convalescent homes and the like. In fact, it has applied the whole resources of medicine to the combat of diseases and the saving of life. The result of this is that at the present thousands of children are born and survive who otherwise would have perished." No doubt some of you saw the statistics of Sir George

Newman on the nation's health, published last month in the daily papers. In this he tells us that in 1900 of the infants under one year 142,912 died; in 1930 only 38,908. As he computed it this was an increase of 40,000 lives each year during the decennium 1921–1930 over and above the decennium 1901–1910. Now although many of these lives saved may prove to be those of efficient citizens, it is equally true that many are inherently defective, mentally and physically. Defectives mate and their families are increasing, while those of the normal people who are the backbone of the nation are decreasing. In fact, as Dr. Tredgold says: "Increased medical knowledge, humanitarian sentiment and social legislation have combined to favour the survival and propagation of the unfit." Such being the case, and there being no pestilences to produce great mortalities, is it surprising that we have a surplus and decadent population to provide work and food for which is the nightmare of all governments? However much the Church, public sentiment and moralists may be against it, I am convinced that in the next generation the question of eugenics must come to the front, and sooner or later some action will be taken to deal with the question of over-population, which invariably leads to unemployment, suffering, pauperism and crime.

And now lastly, with all our increased and actual knowledge of the cause, nature, treatment and prevention of disease, our manifold and more precise methods of diagnosis, why is it, I ask, that concurrently with all this quackery of sundry and divers kind, faith-healing and healing by fancy religions is as rife as it has ever been in the history of the world? There is one thing to be considered, and that is that the subject treated by our profession differs in a material way from that of any other profession. A barrister has to deal with the law, and although he can mould it to make it mean anything he wishes, it cannot answer him back or contradict him. But the subject we have to deal with is a living one, it has a will of its own, a temperament, ever-varying moods, joys, sorrows, emotions, fears, superstitions and a religion. Is it surprising that there are still people in whom we cannot inspire confidence ?

Working on these emotions and superstitions from the beginning of human history and down though all the ages to the present day, quackery has flourished. Just for the sake of comparison with the quackery of to-day I would like to quote an extract from the writings of Rhazes, the famous Arabian physician who lived about 925, with reference to the charlatans of his time. He says : "There are so many arts used by the mountebanks and pretenders to physic that an entire treatise would not contain them. Their impudence is equal to their guilt in tormenting people in their last hours. They profess to cure the falling sickness—epilepsy—by making an issue at the back of the head in the form of a cross. Others give out that they can draw snakes out of their patients' noses or worms from their teeth."

In these days we have osteopathy, chiropraxy, the spiritual healer and the Christian Scientist.[8] As Dr. F. M. R. Walshe states : "It is now not a question of electric currents and magnetism, but of imaginary vertebral subluxation, conveniently too small to be revealed by radiography." "These vertebral subluxations," we are told, "encroach upon the intervertebral foramina and thus interfere with the blood supply to the organs and with the conduction of nervous impulses along the spinal nerves. We are assured by the exponents of this art that all ills our flesh is heir to and all our diseases are simply the various clinical manifestations of vertebral dislocation. Therapy consists in the reduction by manipulation of these abnormalities and generally with a click." I have been told by a manipulator that even paralysis agitans was cured and sleepy sickness is said to be much improved, the trouble being that one vertebra was pressing on the nerves. In one of my cases a woman suffering from recurrent scirrhus in the cervical vertebræ was said by one of these manipulators to have a dislocation of her neck, and this was reduced with a click heard distinctly by her friends all over the room, and they were all convinced (although she died shortly afterwards) that the neck must have been out because they heard such a loud click when it was put back. Another case of persistent sciatica, which had been treated by a specialist in Harley Street, was taken to a manipulator who immediately called attention to the fact that her spine and ribs were out of line, and he proceeded at once to put them straight, and the patient had no doubt about the correctness of his diagnosis because of hearing the grating of the bones as they went into position. But these manipulators do not always confine their attention to the vertebral column, as the following case which came to my knowledge proved. A woman who was suffering from abdominal pain and distention consulted one of these manipulators. He assured her that she had two or three tumours of the womb which he could massage away through the vagina, but it would take some time. Massage of the lower part of the abdomen was accordingly carried out from time to time, she being assured each time that the tumours were getting nearer to the outlet. At length one day the massage was commenced, and he announced that the tumours would soon be delivered, and shortly afterwards he produced two round red bodies and informed the patient that there they were at last! I leave it to you to say who was the more mendacious and blameworthy: the man who in the twentieth century professes to draw two tumours from the womb through the vagina, or the man who in the tenth century asserted that he could draw snakes from his patient's nose and worms from his teeth.

I regret that I have not time to go further into this matter, but I would only say that whilst man is as he is and as described by Southey, "a dupeable animal whose credulity is too strong to resist the impudent assertions of the quack, credulity meaning belief without reason," quackery and faith-healing will still flourish.

I could not close an address of this nature without referring to two innovations which have been of the utmost value to the general practitioners in convenience and the saving of time—the telephone and the motor-car. The motor-car was unreliable at first, and we have heard of practitioners who kept a horse and trap for urgent messages and the motor-car for ordinary work, because if he started in his motor in response to an urgent call he could not rely on reaching his patient without first correcting some engine trouble or mending a puncture. However, in a few years there was a great improvement in motor-cars, and as the late Lord Dewar once said: "Motor-cars were increasing by leaps and bounds and pedestrians survived by the same procedure."

I will only say one or two words in conclusion. What the status of the general practitioner will be in the future one cannot say. Personally, I think specialism will increase, and although I should be very sorry to see it, I fear State Medicine also will, but whatever happens I hope that we shall live up to the traditions of the practitioners who have gone before.[9] No man ever had a higher opinion of his own profession than Sir William Osler, who said that in no other profession is there to be found so large a number of men who have combined intellectual pre-eminence with nobility of character, or who have shown such patient devotion to duty and high ideals. The friend of humanity trusted by rich and poor alike, the physician lives up to the best traditions of an ancient and noble guild. When the work of such a one is over, who can help being reminded of those lines:

"Life's race well run,
Life's work well done,
Life's crown well won,
And after—peace!"

BIBLIOGRAPHY.

[1] McVail, *Brit. Med. Jour.*, 1880, ii. 340.

[2] Power, Sir D'Arcy, *A Short History of Surgery,* Lond., 1933.

[3] Munro Kerr, *Maternal Mortality and Morbidity,* Edinb., 1933.

[4] Newman, *The Rise of Preventive Medicine,* Lond., 1932.

[5] *British Encyclopedia,* "Semmelweiss."

[6] Haggard, *The Lame, Halt and Blind,* New York, 1932.

[7] A. F. Tredgold, "Eugenics," *Camb. Univ. Med. Soc. Magazine,* Easter, 1926.

[8] F. M. R. Walshe, "Some Aspects of Faith Cure," *Camb. Univ. Med. Soc. Magazine,* Lent, 1931.

[9] Osler, Sir William, *Counsels and Ideals,* Oxford, 1905.

